# The complete mitochondrial genome of *Unkanodes sapporonus* (Hemiptera: Delphacidae)

**DOI:** 10.1080/23802359.2019.1692711

**Published:** 2019-11-20

**Authors:** Fang Yu, Ai-Ping Liang

**Affiliations:** a Key Laboratory of Zoological Systematics and Evolution, Institute of Zoology, Chinese Academy of Sciences, Beijing, China;; b College of Life Sciences, University of Chinese Academy of Sciences, Beijing, China

**Keywords:** Delphacidae, mitochondrial genome, phylogenetic relationship

## Abstract

In this study, we sequenced and analyzed the complete mitochondrial genome of *Unkanodes sapporonus* (Matsumura) (Hemiptera: Delphacidae). The mitogenome was 17,765 bp in length with A + T content of 75.87%, containing 13 protein-coding genes, 22 tRNA genes, 2 rRNA genes, and a control region. All protein-coding genes started with ATN, except for *nad5*, which used noncanonical codon GTG. All tRNAs could fold into typical clover-leaf secondary structures, with the exception of *trnS1* (*AGN*).

Many species of Delphacidae (Hemiptera: Fulgoroidea) are economically agricultural pests. Not only do they directly feed on the phloem tissues resulting in serious crop yield losses, but they also transmit plant viruses via sucking. *Unkanodes sapporona* (Matsumura) is a vector of northern cereal mosaic virus (NCMV), rice black-streaked dwarf virus (RBSDV), and rice stripe virus (RSV) (Urban et al. 2010). Here, we sequenced the complete mitochondrial DNA genome of *U. sapporonus* to provide new molecular data to better understand its relationship within the family Delphacidae.

Adults of *U. sapporonus* were collected in Sangzhi County (N 29.69° and E 109.75°), Hunan province, China. The voucher specimen (number F4-030) and its DNA were deposited in the Institute of Zoology, Chinese Academy of Sciences, Beijing, China. Total genomic DNA was extracted using the DNeasy Blood & Tissue Kit (Qiagen, Hilden, Germany), and primers used for mitogenome amplification were modified from those in Yu and Liang (2018). Purified PCR products or multiple clones were sequenced directly. After being assembled, the mitogenome sequence was annotated by the MitoZ software (Meng et al. 2019).

The complete mitochondrial genomes of *U. sapporonus* was 17,765 bp in length (GenBank accession no. MN544774). The mitogenome encodes the entire set of 37 genes (13 protein-coding genes, 22 tRNA genes, and 2 rRNA genes) and a control region, as observed in most insects. The nucleotide composition was A + T biased (75.87%). Gene rearrangement was found in the mitogenome of *U. sapporonus*, congruent with those of other delphacid species such as *Peregrinus maidis* and *Sogatella furcifera*, in which two gene clusters *trnW–trnC–trnY* and *trnT–trnP–nad6* undergo conversion to *trnC–trnW–trnY* and *nad6–trnP–trnT*, respectively (Zhang et al. 2014; Huang and Qin 2017). The canonical start codons ATN were assigned to 12 of all protein-coding genes. The exception was *nad5*, which initiated with GTG. In *U. sapporonus*, the predicted secondary structures of all tRNA genes were typical cloverleaf except for *trnS1* (*AGN*), lacking the dihydrouridine (DHU) stem. The *rrnL* gene was 1206 bp in length with an A + T content of 77.96%, while the *rrnS* gene was 767 bp in size, with a little lower A + T content (74.70%).

There were nine overlaps (23 bp) found in the *U. sapporonus* mitogenome. The *nad4l*–*nad4* overlap was identical to that of *atp8–atp6* (ATGTTAA). A total of 14 intergenic spacers were spread throughout the *U. sapporonus* mitogenome, ranging from 1 to 80 bp. The spacer between *trnS2 (UCN)* and *nad1* was 17 bp in length. As the largest noncoding region, the control region located between *rrnS* and *trnI*, spanning 3319 bp with high A + T content (80.99%).

A maximum-likelihood tree (Figure 1) was inferred from 13 protein-coding genes using the IQ-TREE (Nguyen et al. 2015). We used the meadow spittlebug *Philaenus spumarius* (Hemiptera: Aphrophoridae) as outgroup. In the clade of Delphacinae, *Saccharosydne procerus* was sister to the species of Delphacini. *Unkanodes sapporonus* and *Laodelphax striatellus* were clustered together, indicating their relatively close relationships which were concordant with the previous study based on the multiple loci (Huang et al. 2017).

**Figure 1.**
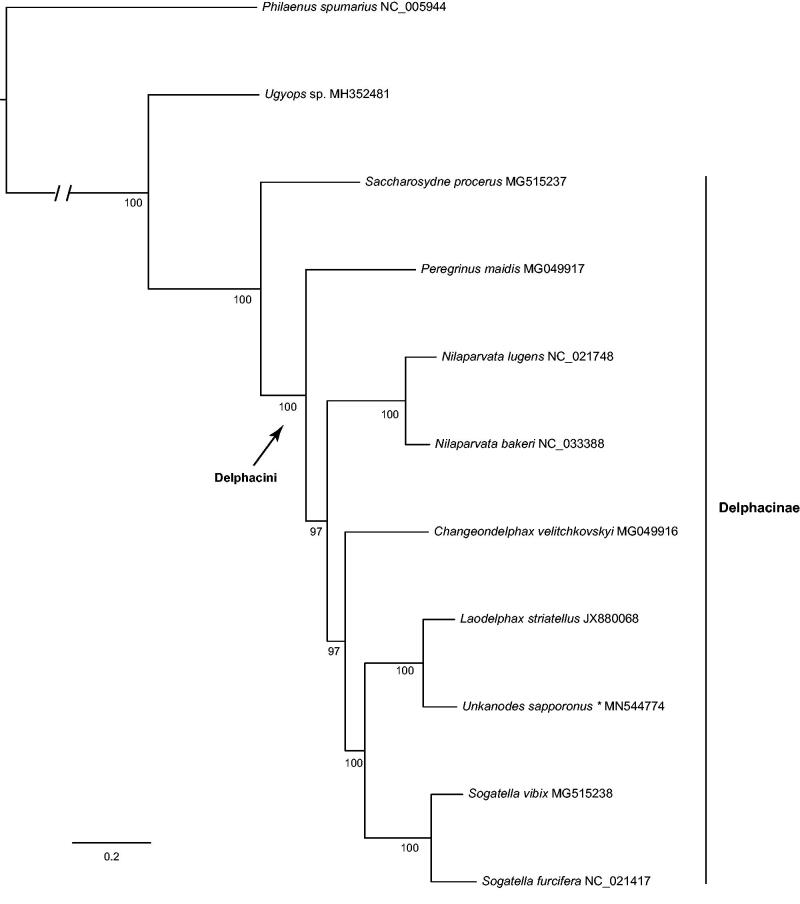
The maximum-likelihood tree of *Unkanodes sapporonus* and other delphacids based on 13 protein-coding genes. Numbers below the branches indicate the bootstrap support values.
